# Self-reported COVID-19 vaccine hesitancy and uptake among participants from different racial and ethnic groups in the United States and United Kingdom

**DOI:** 10.1038/s41467-022-28200-3

**Published:** 2022-02-01

**Authors:** Long H. Nguyen, Amit D. Joshi, David A. Drew, Jordi Merino, Wenjie Ma, Chun-Han Lo, Sohee Kwon, Kai Wang, Mark S. Graham, Lorenzo Polidori, Cristina Menni, Carole H. Sudre, Adjoa Anyane-Yeboa, Christina M. Astley, Erica T. Warner, Christina Y. Hu, Somesh Selvachandran, Richard Davies, Denis Nash, Paul W. Franks, Jonathan Wolf, Sebastien Ourselin, Claire J. Steves, Tim D. Spector, Andrew T. Chan, COPE Consortium

**Affiliations:** 1 Clinical and Translational Epidemiology Unit, Massachusetts General Hospital and Harvard Medical School, Boston, MA USA; 2 Division of Gastroenterology, Massachusetts General Hospital and Harvard Medical School, Boston, MA USA; 3 Department of Biostatistics, Harvard T.H. Chan School of Public Health, Boston, MA USA; 4 Diabetes Unit and Center for Genomic Medicine, Massachusetts General Hospital and Harvard Medical School, Boston, MA USA; 5 Department of Medicine, Massachusetts General Hospital and Harvard Medical School, Boston, MA USA; 6 Broad Institute of MIT and Harvard, Cambridge, MA USA; 7 Department of Epidemiology, Harvard T.H. Chan School of Public Health, Boston, MA USA; 8 School of Biomedical Engineering & Imaging Sciences, King’s College London, London, UK; 9 Zoe Ltd, London, UK; 10 Department of Twin Research and Genetic Epidemiology, King’s College London, London, UK; 11 Computational Epidemiology Lab and Division of Endocrinology, Boston Children’s Hospital and Harvard Medical School, Boston, MA USA; 12 Harvard/MGH Center on Genomics, Vulnerable Populations, and Health Disparities, Massachusetts General Hospital and Harvard Medical School, Boston, MA USA; 13 Institute for Implementation Science in Population Health (ISPH), City University of New York (CUNY), New York, NY USA; 14 Department of Epidemiology and Biostatistics, Graduate School of Public Health and Health Policy, City University of New York (CUNY), New York, NY USA; 15 Department of Clinical Sciences, Lund University, Malmö, Sweden; 16 Department of Immunology and Infectious Disease, Harvard T.H. Chan School of Public Health, Boston, MA USA; 17 Massachusetts Consortium on Pathogen Readiness, Cambridge, MA USA

**Keywords:** Epidemiology, Risk factors, Social sciences

## Abstract

Worldwide, racial and ethnic minorities have been disproportionately impacted by COVID-19 with increased risk of infection, its related complications, and death. In the initial phase of population-based vaccination in the United States (U.S.) and United Kingdom (U.K.), vaccine hesitancy may result in differences in uptake. We performed a cohort study among U.S. and U.K. participants who volunteered to take part in the smartphone-based COVID Symptom Study (March 2020-February 2021) and used logistic regression to estimate odds ratios of vaccine hesitancy and uptake. In the U.S. (*n* = 87,388), compared to white participants, vaccine hesitancy was greater for Black and Hispanic participants and those reporting more than one or other race. In the U.K. (*n* = 1,254,294), racial and ethnic minority participants showed similar levels of vaccine hesitancy to the U.S. However, associations between participant race and ethnicity and levels of vaccine uptake were observed to be different in the U.S. and the U.K. studies. Among U.S. participants, vaccine uptake was significantly lower among Black participants, which persisted among participants that self-reported being vaccine-willing. In contrast, statistically significant racial and ethnic disparities in vaccine uptake were not observed in the U.K sample. In this study of self-reported vaccine hesitancy and uptake, lower levels of vaccine uptake in Black participants in the U.S. during the initial vaccine rollout may be attributable to both hesitancy and disparities in access.

## Abstract (web summary)

The authors show differences in self-reported vaccine hesitancy and uptake among participants from different racial and ethnic groups in the United States and in the United Kingdom during the initial phase of the COVID-19 vaccine rollout.

## Introduction

Severe acute respiratory syndrome coronavirus 2 (SARS-CoV-2) and the COVID-19 pandemic have claimed 4.6 million lives among 221 million confirmed cases worldwide^1^. The speed and urgency with which multiple vaccines have been authorized for use in the United States (U.S.)^2^, the United Kingdom (U.K.)^3–5^, and elsewhere^6^ represent an unrivaled scientific achievement. However, there is a critical need for effective vaccine delivery to realize the promise of ending the pandemic. Logistical hurdles and supply chain difficulties plagued the early phase of a massive global vaccination campaign, particularly in the U.S. While rates have improved considerably in the months since, by February 2021, only 17 doses of vaccine per 100 individuals had been administered in the U.S. compared with 24 per 100 in the U.K^6^.

Racial and ethnic minorities are at particularly increased risk of COVID-19, its related complications, and death^7–9^. Nonetheless, eligibility for most vaccine programs has prioritized health care workers (HCW), older adults and those with comorbidities, but have not considered race or ethnicity^10^. In addition to concerns over fairness and availability, a substantial barrier to uptake in racial and ethnic minority communities is vaccine hesitancy, which may be rooted in ongoing discrimination and prior injustices that have resulted in deeply seated mistrust of the medical system^11,12^.

The U.S. and U.K. have racially and ethnically diverse populations that have been disproportionately affected by the COVID-19 pandemic^7–9^. In contrast to the U.K., which has centralized vaccine delivery and data collection through the National Health Service, initial U.S. efforts were led by fragmented state and local health authorities that had not routinely collected information on race, ethnicity, or vaccine hesitancy^13^ and have not adhered to uniform eligibility criteria^14^. In both countries, there have been reports of racial and ethnic disparities in vaccine uptake, but specific data across a broad community-based sample, particularly in the U.S., are lacking^15–18^.

In this work to assess the real-world impact of the initial phase of these vaccination programs, we use an established smartphone-based data collection tool^19^ to conduct a comparative population-based cohort study to examine country-specific variation in racial and ethnic disparities in vaccine willingness and uptake. We find that racial and ethnic minorities are up to three times as likely to report either being unsure or unwilling to obtain a COVID-19 vaccine, and though some degree of vaccine skepticism is noted among racial and ethnic minority groups in the U.S. and U.K., we observe particularly low vaccine uptake among Black individuals in the U.S., even among those willing to undergo vaccination.

## Results

### Study population

From 24 March 2020 to 1 February 2021, we enrolled a total of 4,797,306 individuals (*n* = 370,282 U.S. participants and *n* = 4,427,024 U.K participants), of whom 1,605,019 individuals were active and logged at least one entry in December 2020 (i.e., 2 weeks prior to the initial vaccine questionnaire). After excluding participants who did not provide information on their racial or ethnic identity and restricting to those who responded to at least one vaccine questionnaire, a final analytic cohort of 1,341,682 individuals remained; Suppl. Fig. 1).

In the U.S., white participants tended to be older and reside in communities with higher income and educational attainment compared to Black or Hispanic participants (Suppl. Methods and Suppl. Table 1). Black and Hispanic participants more frequently reported being a frontline HCW and having previously been infected with SARS-CoV-2. Similar trends were observed among U.K. participants.

### Vaccine hesitancy among racial and ethnic minorities

Among 1,228,638 individuals who answered the question on vaccine willingness, 91% of U.S. participants and 95% of U.K. study participants were willing to accept a COVID-19 vaccine if offered (Suppl. Table 2). In the U.S., participants who were hesitant (unwilling or unsure, respectively) tended to be younger, female, less likely to have had heart disease or cancer, and more likely to live in communities with lower average educational attainment and median incomes. Among frontline HCWs, 7% were unwilling to pursue vaccination and 13% were unsure, compared to 2 and 7%, respectively, across the entire U.S. study population. Similar (younger) age distributions, burden of chronic disease, proportion of frontline HCWs, and rates of prior SARS-CoV-2 infection were observed among U.K. participants.

In both the U.S. and U.K., racial and ethnic minority participants were more likely to report being unsure or unwilling to undergo vaccination. In the U.S., compared to white participants, the age-adjusted ORs for vaccine hesitancy were 3.84 (95% CI: 3.51–4.21) for Black participants, 1.69 (95% CI: 1.53–1.86) for Hispanic participants, and 1.22 (95% CI: 1.03–1.38) for Asian participants, and 2.14 (95% CI: 1.82–2.52) for those who reported other or more than one race (Table 1). Additional adjustment for relevant covariates did not materially alter these risk estimates. We performed a sensitivity analysis to address the possibility of undersampling of certain populations and to assess overall study generalizability by applying a country-level correction for age, sex, and race and ethnicity survey sampling rates using inverse probability weighting (IPW) which demonstrated comparable findings to our primary analyses, though vaccine hesitancy among Asian participants in the U.S. was no longer significantly different from white participants in the U.S. (Suppl. Table 3). Similar degrees of hesitancy were observed among racial and ethnic minorities in the U.K., which was most striking among Black participants (Table 1).

**Table 1:** Vaccine hesitancy by race and ethnicity according to country of enrollment.

	United States				
	White	Black	Hispanic	Asian	More than one/other
Number hesitant or unsure/total	4715/64,144	611/2179	505/3235	309/3089	166/1003
Age-adjusted OR (95% CI)^a^	1.0 (ref.)	3.84 (3.51–4.21)	1.69 (1.53–1.86)	1.22 (1.03–1.38)	2.14 (1.82–2.52)
Multivariable-adjusted OR (95% CI)^b^	1.0 (ref.)	3.68 (3.35–4.05)	1.66 (1.50–1.84)	1.33 (1.17–1.50)	2.13 (1.80–2.52)
Multivariable-adjusted OR (95% CI)^c^	1.0 (ref.)	3.15 (2.86–3.47)	1.42 (1.28–1.58)	1.34 (1.18–1.52)	2.02 (1.70–2.39)

	United Kingdom				
	White	Black	South Asian	Middle East/East Asian	More than one/other
Number hesitant or unsure/total	56,734/1,110,544	1616/8787	1487/15,199	771/6946	1270/13,512
Age-adjusted OR (95% CI)^a^	1.0 (ref.)	3.00 (2.86–3.16)	1.59 (1.51–1.67)	1.83 (1.70–1.97)	1.43 (1.36–1.52)
Multivariable-adjusted OR (95% CI)^b^	1.0 (ref.)	2.96 (2.82–3.12)	1.65 (1.56–1.73)	1.84 (1.71–1.97)	1.42 (1.34–1.50)
Multivariable-adjusted OR (95% CI)^c^	1.0 (ref.)	2.84 (2.69–2.99)	1.66 (1.57–1.76)	1.84 (1.70–1.98)	1.48 (1.39–1.57)

*CI* confidence interval, *OR* odds ratio.

^a^Conditioned upon age and date of study entry.

^b^Additional conditioning upon sex and adjustment for personal history of diabetes, heart disease, lung disease, kidney disease, current smoking status, body mass index, and prior reported history of COVID-19 infection.

^c^Additional adjustment for frontline healthcare worker status, region, and education and income at the community level.

In the U.S., we observed regional differences in willingness to be vaccinated with greater hesitancy in participants in the South (Suppl. Table 4). In the U.K., compared to participants in England, the age-adjusted ORs for vaccine hesitancy was 1.38 (1.25–1.51) for participants in Northern Ireland and 1.10 (1.06–1.15) in Wales. These were not substantially altered after additional adjustment in multivariable models.

When exploring the specific reasons for reluctance, the most frequently indicated concerns among all races and ethnicities related to long-term side effects (50–57%) and adverse reactions (45–54%). Additionally, Black and Hispanic participants cited a lack of knowledge about the vaccine (45–51%) at a higher rate than white participants (37–42%; Suppl. Table 5).

### Racial and ethnic disparities in COVID-19 vaccine uptake

Based on eligibility in the initial phase of mass vaccinations, as expected, vaccinated participants tended to be older, had greater comorbidities, and were considerably more likely to be frontline HCWs (Suppl. Table 6). In the U.S., Black participants were less likely to be vaccinated than white participants (OR 0.71, 95% CI: 0.64–0.79), even after adjusting for age, region, comorbidities, and occupation as a HCW (Table 2). In a subgroup analysis, these associations persisted even when we limited analysis to participants who reported vaccine-willingness (Table 3). In contrast, in the U.K, Black, South Asian, and Middle East and East Asian participants reported lower vaccination rates than white participants in this initial phase of the vaccine rollout, though adjustment for personal and community risk factors attenuated these results. Multivariable risk estimates were comparable after inverse probability weighting (Suppl. Table 3).

**Table 2:** Vaccine uptake by race and ethnicity according to country of enrollment among all participants.

	United States				
	White	Black	Hispanic	Asian	More than one/other
Number receiving a vaccine/total	15,341/64,144	362/2179	519/3235	716/3089	202/1003
Age-adjusted OR (95% CI)^a^	1.0 (ref.)	0.76 (0.68–0.84)	1.00 (0.92–1.10)	1.10 (1.02–1.19)	0.97 (0.84–1.11)
Multivariable-adjusted OR (95% CI)^b^	1.0 (ref.)	0.76 (0.68–0.85)	1.01 (0.93–1.11)	1.07 (0.99–1.15)	0.96 (0.83–1.10)
Multivariable-adjusted OR (95% CI)^c^	1.0 (ref.)	0.71 (0.64–0.79)	0.93 (0.84–1.02)	1.00 (0.93–1.09)	0.94 (0.81–1.08)

	United Kingdom				
	White	Black	South Asian	Middle East/East Asian	More than one/other
Number receiving a vaccine/total	171,453/1,110,544	1022/8787	2339/15,199	922/6946	1506/13,512
Age-adjusted OR (95% CI)^a^	1.0 (ref.)	1.13 (1.06–1.20)	1.34 (1.29–1.40)	1.10 (1.03–1.18)	1.05 (1.00–1.11)
Multivariable-adjusted OR (95% CI)^b^	1.0 (ref.)	1.12 (1.06–1.19)	1.31 (1.25–1.36)	1.09 (1.02–1.16)	1.04 (0.98–1.09)
Multivariable-adjusted OR (95% CI)^c^	1.0 (ref.)	0.98 (0.92–1.04)	1.18 (1.13–1.23)	1.01 (0.94–1.08)	0.99 (0.93–1.04)

Data are shown through 1 February 2021.

*CI* confidence interval, *OR* odds ratio.

^a^Conditioned upon age and date of study entry.

^b^Additional conditioning upon sex and adjustment for personal history of diabetes, heart disease, lung disease, kidney disease, current smoking status, body mass index, and prior reported history of COVID-19 infection.

^c^Additional adjustment for frontline healthcare worker status, region, and education and income at the community level.

**Table 3:** Vaccine uptake by race and ethnicity according to country of enrollment among the vaccine-willing.

	United States				
	White	Black	Hispanic	Asian	More than one/other
Number receiving a vaccine/total	15,062/59,429	328/1568	499/2730	681/2780	192/837
Age-adjusted OR (95% CI)^a^	1.0 (ref.)	0.88 (0.78–0.98)	1.04 (0.95–1.14)	1.11 (1.03–1.20)	1.03 (0.89–1.19)
Multivariable-adjusted OR (95% CI)^b^	1.0 (ref.)	0.88 (0.78–0.98)	1.05 (0.96–1.15)	1.08 (1.00–1.17)	1.02 (0.88–1.17)
Multivariable-adjusted OR (95% CI)^c^	1.0 (ref.)	0.82 (0.73–0.92)	0.95 (0.86–1.04)	1.01 (0.93–1.09)	1.00 (0.86–1.16)

	United Kingdom				
	White	Black	South Asian	Middle East/East Asian	More than one/other
Number receiving a vaccine/total	168,369/1,053,810	951/7171	2255/13,712	884/6175	1469/12,242
Age-adjusted OR (95% CI)^a^	1.0 (ref.)	1.22 (1.14–1.30)	1.37 (1.31–1.42)	1.13 (1.06–1.21)	1.09 (1.03–1.15)
Multivariable-adjusted OR (95% CI)^b^	1.0 (ref.)	1.22 (1.14–1.30)	1.33 (1.28–1.39)	1.12 (1.05–1.20)	1.07 (1.01–1.13)
Multivariable-adjusted OR (95% CI)^c^	1.0 (ref.)	1.07 (1.00–1.14)	1.21 (1.16–1.26)	1.04 (0.97–1.11)	1.02 (0.97–1.08)

Data are shown through 1 February 2021.

*CI* confidence interval, *OR* odds ratio.

^a^Conditioned upon age and date of study entry.

^b^Additional conditioning upon sex and adjustment for personal history of diabetes, heart disease, lung disease, kidney disease, current smoking status, body mass index, and prior reported history of COVID-19 infection.

^c^Additional adjustment for frontline healthcare worker status, region, and education and income at the community level.

**Fig. 1:**
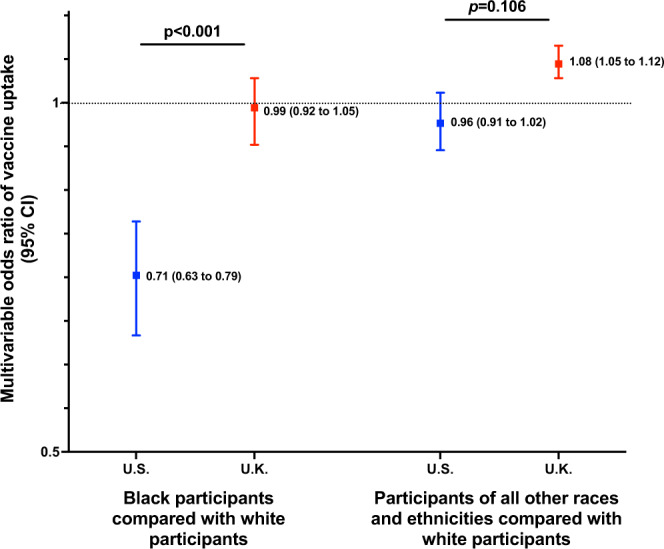
Disparity in vaccine uptake by race and ethnicity according to country of enrollment. Risk estimates of receiving a vaccine through 1 February 2021 calculated within the country using multivariable logistic regression conditioned upon age, sex, and date of study entry and adjusted for personal history of diabetes, heart disease, lung disease, kidney disease, current smoking status, body mass index, prior reported history of COVID-19 infection, frontline healthcare worker status, and education and income at the community level. Data are presented as multivariable OR estimates ±95% CI. *P*_interaction_ was calculated using the Wald test for the cross-product terms between race and ethnicity and country, *P*_interaction_ = for Black vs. white and 0.106 for all other races and ethnicities compared with white participants, respectively. *N* = 1,110,544 for white U.K. participants, 64,144 for white U.S. participants, 8787 for Black U.K. participants, 2179 for Black U.S. participants, 35,657 U.K. participants of other races and ethnicities, and 7327 U.S. participants of other races and ethnicities, respectively. Source data are provided with this paper. CI confidence interval, OR odds ratio.

The disparity in vaccine uptake among Black participants compared with white participants differed significantly by country of study (*P*_heterogeneity_ < 0.001). When compared to white participants within their respective countries, Black participants were less likely to be vaccinated in the U.S. compared with Black participants in the U.K. (Fig. 1). Compared to the Northeast of the U.S., vaccine uptake was comparatively greater in other parts of the U.S. In the U.K., England appeared to have greater levels of vaccine uptake compared to other countries where increased vaccine hesitancy has been documented (Suppl. Table 7)^20^.

Vaccine uptake among Black participants in the U.S. study was comparable among specific sociodemographic groups, including frontline HCWs (Table 4). Notably, in the U.K. study, Black participants that were frontline HCWs had lower vaccine uptake than their white counterparts (Table 5). Black U.S. participants living in communities with lower educational attainment also had lower vaccine uptake (Table 6). Finally, no consistent differences were observed in localized vaccine symptoms (e.g., pain or swelling among others) according to race and ethnicity (Suppl. Table 8).

**Table 4:** Vaccine uptake by race and ethnicity according to country of enrollment among frontline healthcare workers and the general community.

	United States				
	White	Black	Hispanic	Asian	More than one/other
*Frontline healthcare workers*					
Number receiving a vaccine/total	827/1906	28/112	41/136	41/89	18/44
Age-adjusted OR (95% CI)^a^	1.0 (ref.)	0.59 (0.38–0.92)	0.85 (0.57–1.27)	1.00 (0.64–1.55)	1.01 (0.54–1.90)
Multivariable-adjusted OR (95% CI)^b^	1.0 (ref.)	0.63 (0.39–1.03)	0.81 (0.52–1.25)	1.11 (0.68–1.80)	1.02 (0.50–2.06)
Multivariable-adjusted OR (95% CI)^c^	1.0 (ref.)	0.67 (0.40–1.11)	0.84 (0.53–1.33)	1.01 (0.62–1.66)	1.04 (0.51–2.10)
*General community*					
Number receiving a vaccine/total	14,514/62,238	334/2067	478/3099	675/3000	184/959
Age-adjusted OR (95% CI)^a^	1.0 (ref.)	0.75 (0.68–0.84)	1.01 (0.92–1.11)	1.10 (1.01–1.18)	0.95 (0.82–1.10)
Multivariable-adjusted OR (95% CI)^b^	1.0 (ref.)	0.76 (0.68–0.85)	1.02 (0.93–1.12)	1.07 (0.98–1.15)	0.94 (0.81–1.09)
Multivariable-adjusted OR (95% CI)^c^	1.0 (ref.)	0.73 (0.65–0.82)	0.93 (0.85–1.03)	0.99 (0.91–1.08)	0.91 (0.79–1.06)

	United Kingdom				
	White	Black	South Asian	Middle East/East Asian	More than one/other
*Frontline healthcare workers*					
Number receiving a vaccine/total	14,955/25,449	158/373	313/579	106/204	152/320
Age-adjusted OR (95% CI)^a^	1.0 (ref.)	0.70 (0.59–0.82)	0.94 (0.84–1.06)	0.87 (0.72–1.06)	0.79 (0.67–0.93)
Multivariable-adjusted OR (95% CI)^b^	1.0 (ref.)	0.71 (0.6–0.84)	0.97 (0.86–1.10)	0.85 (0.70–1.05)	0.81 (0.68–0.96)
Multivariable-adjusted OR (95% CI)^c^	1.0 (ref.)	0.71 (0.6–0.85)	0.95 (0.84–1.09)	0.85 (0.68–1.05)	0.78 (0.65–0.94)
*General community*					
Number receiving a vaccine/total	156,498/1,085,095	864/8414	2026/14,620	816/6742	1354/13,192
Age-adjusted OR (95% CI)^a^	1.0 (ref.)	1.13 (1.06–1.21)	1.34 (1.28–1.4)	1.11 (1.04–1.19)	1.10 (1.04–1.16)
Multivariable-adjusted OR (95% CI)^b^	1.0 (ref.)	1.13 (1.05–1.21)	1.30 (1.24–1.36)	1.10 (1.02–1.18)	1.08 (1.02–1.14)
Multivariable-adjusted OR (95% CI)^c^	1.0 (ref.)	1.05 (0.98–1.13)	1.22 (1.17–1.28)	1.05 (0.98–1.13)	1.02 (0.97–1.08)

Data are shown through 1 February 2021.

*CI* confidence interval, *OR* odds ratio.

^a^Conditioned upon age and date of study entry.

^b^Additional conditioning upon sex and adjustment for personal history of diabetes, heart disease, lung disease, kidney disease, current smoking status, body mass index, and prior reported history of COVID-19 infection.

^c^Additional adjustment for frontline healthcare worker status, region, and education and income at the community level (except in models for given strata).

**Table 5:** Vaccine uptake by race and ethnicity according to country of enrollment by economic deprivation.

	United States				
	White	Black	Hispanic	Asian	More than one/other
*Lower income (Quartile 1)*					
Number receiving a vaccine/total	3660/15,207	156/937	152/1033	71/421	55/267
Age-adjusted OR (95% CI)^a^	1.0 (ref.)	0.73 (0.62–0.86)	0.99 (0.84–1.18)	0.90 (0.71–1.15)	1.13 (0.85–1.49)
Multivariable-adjusted OR (95% CI)^b^	1.0 (ref.)	0.75 (0.63–0.89)	0.99 (0.83–1.19)	0.89 (0.69–1.14)	1.07 (0.80–1.43)
Multivariable-adjusted OR (95% CI)^c^	1.0 (ref.)	0.73 (0.61–0.86)	0.94 (0.79–1.14)	0.90 (0.70–1.16)	1.08 (0.81–1.44)
*Higher income (Quartile 4)*					
Number receiving a vaccine/total	3645/15,599	54/302	115/630	244/1089	53/225
Age-adjusted OR (95% CI)^a^	1.0 (ref.)	0.86 (0.65–1.13)	1.13 (0.93–1.36)	1.09 (0.95–1.24)	1.13 (0.85–1.50)
Multivariable-adjusted OR (95% CI)^b^	1.0 (ref.)	0.85 (0.64–1.13)	1.15 (0.94–1.39)	1.08 (0.94–1.24)	1.12 (0.84–1.49)
Multivariable-adjusted OR (95% CI)^c^	1.0 (ref.)	0.83 (0.62–1.11)	1.05 (0.86–1.28)	0.97 (0.84–1.12)	1.09 (0.81–1.45)

	United Kingdom				
	White	Black	South Asian	Middle East/East Asian	More than one/other
*Lower income (Quartile 1)*					
Number receiving a vaccine/total	41,761/283,212	455/3904	745/5377	289/2260	459/4524
Age-adjusted OR (95% CI)^a^	1.0 (ref.)	1.12 (1.02–1.23)	1.24 (1.15–1.33)	1.15 (1.02–1.29)	0.97 (0.88–1.06)
Multivariable-adjusted OR (95% CI)^b^	1.0 (ref.)	1.11 (1.01–1.22)	1.21 (1.12–1.30)	1.15 (1.02–1.29)	0.95 (0.87–1.04)
Multivariable-adjusted OR (95% CI)^c^	1.0 (ref.)	0.97 (0.89–1.07)	1.12 (1.04–1.21)	1.05 (0.94–1.18)	0.90 (0.82–0.99)
*Higher income (Quartile 4)*					
Number receiving a vaccine/total	29,993/192,710	111/863	345/2250	136/1063	224/2018
Age-adjusted OR (95% CI)^a^	1.0 (ref.)	1.18 (0.98–1.42)	1.32 (1.19–1.47)	0.99 (0.83–1.17)	1.04 (0.91–1.19)
Multivariable-adjusted OR (95% CI)^b^	1.0 (ref.)	1.20 (1.00–1.45)	1.29 (1.16–1.43)	0.97 (0.81–1.14)	1.03 (0.90–1.18)
Multivariable-adjusted OR (95% CI)^c^	1.0 (ref.)	1.08 (0.90–1.31)	1.19 (1.07–1.33)	0.91 (0.77–1.08)	1.01 (0.88–1.15)

Data are shown through 1 February 2021.

*CI* confidence interval, *OR* odds ratio.

^a^Conditioned upon age and date of study entry.

^b^Additional conditioning upon sex and adjustment for personal history of diabetes, heart disease, lung disease, kidney disease, current smoking status, body mass index, and prior reported history of COVID-19 infection.

^c^Additional adjustment for frontline healthcare worker status, region, and education and income at the community level (except in models for a given strata).

**Table 6:** Vaccine uptake by race and ethnicity according to country of enrollment by educational attainment.

	United States				
	White	Black	Hispanic	Asian	More than one/other
*Lower educational attainment (Quartile 1)*					
Number receiving a vaccine/total	3441/14,875	141/926	189/1221	165/610	55/304
Age-adjusted OR (95% CI)^a^	1.0 (ref.)	0.69 (0.58–0.82)	1.11 (0.95–1.30)	1.18 (1.01–1.39)	0.91 (0.69–1.21)
Multivariable-adjusted OR (95% CI)^b^	1.0 (ref.)	0.70 (0.58–0.83)	1.15 (0.98–1.36)	1.14 (0.96–1.35)	0.87 (0.66–1.16)
Multivariable-adjusted OR (95% CI)^c^	1.0 (ref.)	0.65 (0.54–0.78)	1.06 (0.90–1.26)	1.10 (0.92–1.31)	0.86 (0.64–1.14)
*Higher educational attainment (Quartile 4)*					
Number receiving a vaccine/total	3808/16,031	56/278	88/528	155/800	46/214
Age-adjusted OR (95% CI)^a^	1.0 (ref.)	0.96 (0.73–1.26)	1.03 (0.83–1.28)	1.27 (1.07–1.50)	1.06 (0.78–1.44)
Multivariable-adjusted OR (95% CI)^b^	1.0 (ref.)	0.97 (0.73–1.28)	1.05 (0.84–1.32)	1.28 (1.08–1.52)	1.06 (0.78–1.45)
Multivariable-adjusted OR (95% CI)^c^	1.0 (ref.)	0.93 (0.70–1.24)	0.95 (0.75–1.19)	1.19 (1.00–1.42)	1.09 (0.80–1.50)

	United Kingdom				
	White	Black	South Asian	Middle East/East Asian	More than one/other
*Lower educational attainment (Quartile 1)*					
Number receiving a vaccine/total	39,534/273,555	354/2822	439/3422	178/1324	289/2881
Age-adjusted OR (95% CI)^a^	1.0 (ref.)	1.22 (1.10–1.36)	1.14 (1.03–1.25)	1.16 (1.00–1.35)	0.94 (0.84–1.05)
Multivariable-adjusted OR (95% CI)^b^	1.0 (ref.)	1.21 (1.09–1.34)	1.13 (1.03–1.24)	1.18 (1.02–1.37)	0.93 (0.83–1.04)
Multivariable-adjusted OR (95% CI)^c^	1.0 (ref.)	1.08 (0.97–1.20)	1.06 (0.97–1.17)	1.09 (0.94–1.27)	0.94 (0.83–1.05)
*Higher educational attainment (Quartile 4)*					
Number receiving a vaccine/total	35,969/217,167	153/1259	503/3215	220/1632	346/3110
Age-adjusted OR (95% CI)^a^	1.0 (ref.)	1.07 (0.91–1.25)	1.30 (1.19–1.43)	1.05 (0.92–1.20)	1.03 (0.93–1.14)
Multivariable-adjusted OR (95% CI)^b^	1.0 (ref.)	1.06 (0.90–1.24)	1.27 (1.16–1.39)	1.02 (0.89–1.17)	1.01 (0.91–1.13)
Multivariable-adjusted OR (95% CI)^c^	1.0 (ref.)	0.96 (0.81–1.12)	1.13 (1.03–1.23)	0.93 (0.81–1.06)	0.96 (0.86–1.07)

Data are shown through 1 February 2021.

*CI* confidence interval, *OR* odds ratio.

^a^Conditioned upon age and date of study entry.

^b^Additional conditioning upon sex and adjustment for personal history of diabetes, heart disease, lung disease, kidney disease, current smoking status, body mass index, and prior reported history of COVID-19 infection.

^c^Additional adjustment for frontline healthcare worker status, region, and education and income at the community level (except in models for given strata).

## Discussion

Among 1,341,682 participants in the U.S. and U.K., we observed increased COVID-19 vaccine hesitancy among racial and ethnic minority participants. Further, through the early phase of each country’s mass vaccination campaign (data through 1 February 2021), we revealed significant racial and ethnic disparities in uptake in the U.S., but not the U.K., even among the vaccine-willing, suggesting issues related to access may underlie the observed lower vaccine uptake among minority populations in the U.S. Interestingly, we observed a higher than anticipated rate of vaccine hesitancy among frontline HCWs, perhaps due to their substantially higher rate of prior COVID-19 infection^21^ and heightened concern about safety.

Our findings of greater vaccine hesitancy among minority participants confirm findings from prior investigations with smaller sample sizes^22–24^. Deep-rooted and ongoing mistrust of the medical system among people of color^25^ and a lack of diverse representation in clinical trials^26,27^ may play a role in explaining this hesitancy. Moreover, racial and ethnic minorities who have already borne the disproportionate brunt of the pandemic^28,29^ may have been taking a more cautious approach to new vaccines. Our data did not reveal differences in self-reports of localized injection-site reactions by race or ethnicity. Prior work specifically examining attitudes toward COVID-19 vaccines further support our findings. A recent randomized controlled trial demonstrated that COVID-19 vaccine misinformation significantly reduced vaccination intent in the U.K. and U.S^30^. Notably, in that study, differences in susceptibility and receptiveness were observed across sociodemographic groups.

Our results demonstrating lower early vaccine uptake among communities of color have been shown in other studies, though uptake has improved somewhat in the latter phase of the vaccine rollout^31,32^. A recent study of U.K. HCWs showed substantially lower vaccine uptake among racial and ethnic minorities^33^. Our results extend these data by concurrently examining vaccine hesitancy and vaccine uptake within the same participants from community-based samples in two countries. We found that even among the vaccine-willing participants in the US with access to smartphone technology in the early phases of the mass vaccination campaigns, Black participants were less likely to receive a vaccine, whereas in the U.K. study, no consistent disparities in vaccine uptake were observed.

The strengths of our study include the prospective population-scale enrollment of a diverse group of participants from two comparably afflicted nations using a common data collection instrument. With disparate approaches to COVID-19 vaccination campaigns and healthcare delivery in the U.S. and U.K., our multinational study design provided a unique opportunity to consider the degree to which structural inequities, public mistrust, and unequal care access could result in differences in vaccine willingness and uptake. Our use of a digital platform to rapidly collect this information on vaccine skepticism and usage provides real-time actionable insights to inform the public health response to an ongoing pandemic. Finally, extensive demographic and comorbidity information are generally not available in registry-level data or large-scale surveillance efforts, and we had an opportunity to evaluate whether these established risk factors could influence vaccine attitudes and uptake.

We acknowledge several limitations to this study. We relied primarily on volunteered information which may be subject to measurement and reporting bias. However, our validation study (Suppl. Methods) demonstrates that self-reported information from the general population was accurately and faithfully reported, and prior studies investigating the accuracy of self-reported influenza vaccination demonstrated >93% agreement against an immunization registry, even when queried against the prior flu season^34^. While our study had comparatively lower proportions of racial and ethnic minority participants, we enrolled relatively high absolute numbers of participants for most demographic groups, and sensitivity analyses employing inverse probability-based methods to downweigh oversampled respondents did not materially alter our primary findings. Despite similar recruitment strategies of both the general public and participants of long-running cohort studies, the enrolled population in the U.K. was much larger than its U.S. counterpart. However, our sample size remained substantial in the U.S., which still allowed for internally consistent country-specific estimates. To maximize participation and to balance privacy concerns among vulnerable populations, greater detail on racial and ethnic self-identity or individual-level data on education and income could not be obtained, and our current categorizations may oversimplify or incompletely characterize the different lived experiences of participants of racial and ethnic minorities navigating the healthcare system.

Despite more than 80% of U.S. adults adopting smartphones^35^, we acknowledge that our data collection may have comparatively lower penetrance among certain socioeconomic/age groups. However, under-recruitment of more socioeconomically disadvantaged or less technologically literate participants may have led to different results. In fact, the use of a smartphone application for data collection allowed us to highlight racial and ethnic disparities in uptake that persisted despite uniform access to technology. Finally, our cohort of study volunteers willing to share information about COVID-19 does not represent a random sampling of the U.S. and U.K. population and are likely enriched for individuals that are generally more accepting of vaccinations. Nonetheless, the differences we observed in vaccine hesitancy and uptake among participants of different racial and ethnic groups remain internally valid and likely underestimate broader disparities within population samples that do not use a common data collection instrument or rely on voluntary participation.

We found significantly higher likelihood of COVID-19 vaccine hesitancy among racial and ethnic minority groups in the U.S. and the U.K., particularly among Black and Hispanic participants, supporting the need for targeted vaccine education from trusted messengers. Furthermore, during the early phases of the mass vaccination campaigns in the U.S., a statistically significant disparity in vaccine uptake among Black participants was observed. This statistically significant difference, not found in the U.K. study, suggests that initial disparities in U.S. vaccine uptake could have been exacerbated by inequities in the prioritization and distribution of vaccines to minority communities in the U.S. Given the relative lack of a national public health infrastructure in the U.S., our data highlight the potential value of a more centralized system of vaccine delivery to facilitate more equitable uptake in the initial phase of a vaccine rollout. Of course, even a more centralized system cannot ensure fully equitable delivery given the inherent variation in health care services at the local level. Taken together, these findings support the need to address long-standing systemic disparities to achieve the health equity required for population-scale immunity.

## Methods

### Study design and participants

We performed a cohort study in the U.S. and U.K. using the COVID Symptom Study (CSS) smartphone application developed by Zoe Ltd. in collaboration with researchers at the Massachusetts General Hospital, King’s College London, Lund University, and Uppsala University^19^. The CSS application was designed to capture information for an observational trial on potential COVID-19 symptoms (ClinicalTrials.gov registration NCT04331509) and was later adapted to collect data on additional unregistered outcomes, including vaccine hesitancy and vaccine uptake. At enrollment, participants aged 18 years or greater provided informed consent to the use of volunteered information for research and agreed to applicable privacy policies and terms of use. This research study was approved by the Mass General Brigham Human Research Committee (Institutional Review Board Protocol 2020P000909) and King’s College London Ethics Committee (REMAS ID 18210).

Beginning 10 December 2020–2 days after the first authorized vaccine administration to a member of the U.K. public^36^–we introduced a questionnaire to U.K. participants assessing whether they received a vaccine dose. Starting 7 January 2021 in the U.S., we collected the same information on vaccine uptake, as well as participant willingness to obtain a COVID-19 vaccine (yes/no/unsure) if they were not yet vaccinated, as well as any suspected vaccine-related symptoms. For those unsure about or unwilling to receive a vaccine, we queried their underlying reasons (Suppl. Table 9). At the time the revised vaccine questionnaire was launched in the U.S., the U.K.-based questionnaire was updated with new vaccine-related questions pushed to all active users, including those who had responded to the prior vaccine questionnaire.

### Ascertainment of racial and ethnic identity

Information collected using the CSS application has previously been provided^19^. Briefly, at download and study enrollment, participants were asked to provide baseline demographic information, as well as details on suspected risk factors or relevant comorbidities (Table 1). They were asked with which race and/or ethnicity they self-identified based on standardized categories from the National Institutes of Health (NIH) in the U.S. and the Office for National Statistics in the U.K. (Suppl. Table 10 and Suppl. Methods)^37,38^. In the U.S., Hispanic classification was defined as any race of Hispanic or Latino ancestry. Non-Hispanic categories were defined as each respective race not of Hispanic or Latino ancestry. Responses were then aggregated in a manner consistent with prior analyses^29^. We excluded individuals who selected “Prefer not to say” as their response or did not answer these questions.

### Ascertainment of other covariates and exposures

We collected information on age (years), sex at birth (male, female, or other), weight (kg), and height (meters) were used to calculate body mass index (BMI, < 18.5, 18.5–24.9, 25–29.9, and ≥30 kg/m^2^), prior history of diabetes, heart disease, lung disease, kidney disease, or active malignancy (each yes/no), smoking history (current/prior vs. never), and frontline HCW status (yes/no). We longitudinally ascertained whether they had ever tested positive for COVID-19 (yes/no), which was previously shown to have an excellent agreement between self-report and confirmed test reports (Suppl. Methods).

### Statistical analysis

To investigate determinants of COVID-19 vaccine hesitancy and uptake, we performed multivariable logistic regression to estimate odds ratios (OR) and their 95% confidence intervals (CIs) conditioned upon age, sex, and date of study entry adjusting for history of diabetes, heart disease, lung disease, kidney disease, cancer, current/prior smoking status, BMI, prior history of COVID-19 infection, occupation as frontline HCW, geographic region (U.S.) or country (U.K.), and sociodemographic factors based on community-level measures of educational and financial deprivation (Suppl. Methods). To address the possibility of undersampling and to more robustly assess the generalizability of our survey results, we conducted inverse probability weighting (IPW) analyses^39,40^ to examine whether applying country-specific census-level correction for age, sex, and race and ethnicity sampling rates influenced our primary findings. We performed stratified analyses among frontline HCWs and the general community, and both lower and higher community-level educational attainment and financial deprivation, respectively. Formal tests for interaction were assessed using the Wald test in models with country-by-race and ethnicity interaction terms. Finally, we reported the prevalence of localized injection-site symptoms among vaccinated participants. Two-sided *p*-values <0.05 were considered statistically significant. All statistical analyses were performed using R 4.0.3 (Vienna, Austria) and packages from the Bioconductor 3.12 release.

### Reporting summary

Further information on research design is available in the Nature Research Reporting Summary linked to this article.

## Supplementary information

Supplementary Information

Reporting Summary

## Data Availability

All study authors had access to participant-level data. To comply with informed participant consent, which stipulated raw anonymized individual-level data would only be available upon request for research purposes, data collected using the COVID Symptom Study smartphone application are being shared with other researchers through the U.K. National Health Service-funded Health Data Research UK (HDRUK) and Secure Anonymised Information Linkage consortium, housed in the U.K. Secure Research Platform (Swansea, UK, https://web.www.healthdatagateway.org/dataset/fddcb382-3051-4394-8436-b92295f14259). U.S. investigators are encouraged to coordinate data requests through the Coronavirus Pandemic Epidemiology (COPE) Consortium (Suppl. Table 11, https://www.monganinstitute.org/cope-consortium). Source data are provided with this paper.

## Source data

Source Data

## Code availability

No custom code was used for data analysis. A package for COVID Symptom Study data extraction and wrangling is freely available at https://github.com/KCL-BMEIS/ExeTera/.
