# Author Correction: Self-reported COVID-19 vaccine hesitancy and uptake among participants from different racial and ethnic groups in the United States and United Kingdom

**DOI:** 10.1038/s41467-022-29100-2

**Published:** 2022-03-25

**Authors:** Long H. Nguyen, Amit D. Joshi, David A. Drew, Jordi Merino, Wenjie Ma, Chun-Han Lo, Sohee Kwon, Kai Wang, Mark S. Graham, Lorenzo Polidori, Cristina Menni, Carole H. Sudre, Adjoa Anyane-Yeboa, Christina M. Astley, Erica T. Warner, Christina Y. Hu, Somesh Selvachandran, Richard Davies, Denis Nash, Paul W. Franks, Jonathan Wolf, Sebastien Ourselin, Claire J. Steves, Tim D. Spector, Andrew T. Chan, COPE Consortium

**Affiliations:** 1 Clinical and Translational Epidemiology Unit, Massachusetts General Hospital and Harvard Medical School, Boston, MA USA; 2 Division of Gastroenterology, Massachusetts General Hospital and Harvard Medical School, Boston, MA USA; 3 Department of Biostatistics, Harvard T.H. Chan School of Public Health, Boston, MA USA; 4 Diabetes Unit and Center for Genomic Medicine, Massachusetts General Hospital and Harvard Medical School, Boston, MA USA; 5 Department of Medicine, Massachusetts General Hospital and Harvard Medical School, Boston, MA USA; 6 Broad Institute of MIT and Harvard, Cambridge, MA USA; 7 Department of Epidemiology, Harvard T.H. Chan School of Public Health, Boston, MA USA; 8 School of Biomedical Engineering & Imaging Sciences, King’s College London, London, UK; 9 Zoe Ltd, London, UK; 10 Department of Twin Research and Genetic Epidemiology, King’s College London, London, UK; 11 Computational Epidemiology Lab and Division of Endocrinology, Boston Children’s Hospital and Harvard Medical School, Boston, MA USA; 12 Harvard/MGH Center on Genomics, Vulnerable Populations, and Health Disparities, Massachusetts General Hospital and Harvard Medical School, Boston, MA USA; 13 Institute for Implementation Science in Population Health (ISPH), City University of New York (CUNY), New York, NY USA; 14 Department of Epidemiology and Biostatistics, Graduate School of Public Health and Health Policy, City University of New York (CUNY), New York, NY USA; 15 Department of Clinical Sciences, Lund University, Malmö, Sweden; 16 Department of Immunology and Infectious Disease, Harvard T.H. Chan School of Public Health, Boston, MA USA; 17 Massachusetts Consortium on Pathogen Readiness, Cambridge, MA USA

**Keywords:** Epidemiology, Risk factors, Social sciences

Correction to: *Nature Communications* 10.1038/s41467-022-28200-3, published online 1 February 2022.

The original version of this Article contained an error in Table 1. The value listed for ‘Number hesitant or unsure/total’ in the column labelled ‘White’ under the subheading ‘United Kingdom’ incorrectly read ‘567,734/1,110,544’ instead of ‘56,734/1,110,544’. This has been corrected in both the PDF and HTML versions of the Article.

