# Supplementary material for: Self-reported COVID-19 vaccine hesitancy and uptake among participants from different racial and ethnic groups in the United States and United Kingdom
Source: Nat Commun. 2022 Feb 1;13:636. doi: 10.1038/s41467-022-28200-3 (PMC8807721; doi:10.1038/s41467-022-28200-3)
Supplement: Supplementary file 1 — Supplementary Information [file 41467_2022_28200_MOESM1_ESM.pdf]

Self-reported COVID-19 vaccine hesitancy and uptake among participants from different racial and ethnic groups in the United States and United Kingdom

Long H. Nguyen*, Amit D. Joshi*, David A. Drew*, Jordi Merino, Wenjie Ma, Chun-Han Lo, Sohee Kwon, Kai Wang, Mark S. Graham, Lorenzo Polidori, Cristina Menni, Carole H. Sudre, Adjoa Anyane-Yeboa, Christina M. Astley, Erica T. Warner, Christina Y. Hu, Somesh Selvachandran, Richard Davies, Denis Nash, Paul W. Franks, Jonathan Wolf, Sebastien Ourselin, Claire J. Steves, Tim D. Spector, and Andrew T. Chan, COPE Consortium
*equal contribution
A list of all contributing consortium authors can be found at the end of the document

Supplementary Information

Supplementary Methods **2**

Community-level sociodemographic factors 2

Supplementary statistical analysis, handling of missing data, and power 3

Supplementary Figures & Tables **4**

Supplementary Figure 1. Study diagram 4

Supplementary Table 1. Baseline characteristics of study participants by race and ethnicity according to country of enrollment 5

Supplementary Table 2. Baseline characteristics of study participants by vaccine willingness and country of enrollment 9

Supplementary Table 3. Inverse probability weighted analyses of vaccine hesitancy and uptake by race and ethnicity according to country of enrollment 12

Supplementary Table 4. Vaccine hesitancy by geographical region according to country of enrollment 14

Supplementary Table 5. Reasons for vaccine hesitancy by race and ethnicity according to country of enrollment 15

Supplementary Table 6. Baseline characteristics of study participants by vaccination status and country of enrollment 17

Supplementary Table 7. Vaccine uptake by geographical region according to country of enrollment 20

Supplementary Table 8. Localized symptoms among vaccinated participants according to country of enrollment 21

Supplementary Table 9. Vaccine-related questions through February 1, 2021 22

Supplementary Table 10. Race and ethnicity categories by country of enrollment 24

Supplementary Table 11. Partnering cohorts within the COPE Consortium 25

Supplementary References **26**

Supplementary Methods

Prior validation work

To ascertain the validity of self-reported COVID Symptom Study information, we performed a validation study among a random subset of 235 users who reported a COVID-19 test from whom we requested photographs of their COVID-19 test reports. Study staff blinded to participant-provided information found excellent agreement between self-report and test reports with 88% sensitivity and 94% specificity.

Ascertainment of racial and ethnic identity

Individuals who identified their race or ethnicity as "Other" were offered an opportunity to provide a free-text entry. Those who identified as "Mixed Race" or selected more than one race were categorized as "More than one race". "Native Hawaiian and Pacific Islanders" were classified as Asian. Due to limited sample sizes, "American Indian or Alaskan Natives" were categorized as "Other", and "Other" and "More than one race" were combined. In the U.K., individuals were asked whether they identified as "Chinese" or "Asian/British Asian", which offered the following examples, but did not specifically ask about other racial identities from the Asian continent: "Indian, Pakistani, Bangladeshi, Other".

Community-level sociodemographic factors

Participants who elected to share information on their zip code (U.S./U.K.) or Lower Layer Super Output Area (LSOA, U.K.) of residence were assigned community-level socioeconomic measures. Socioeconomic measures were generated using established metrics derived from aggregated census data: proportion of individuals aged ≥25 years old with a Bachelor's degree and median annual income (U.S.)[1] and the education and income measures from the U.K.'s Indices of Multiple Deprivation (deciles, IMD),[2] respectively. U.S. zip codes were further categorized into broader regions using U.S. Census Bureau criteria[3] (American Northeast,

Midwest, South, and West) and LSOAs were linked to one of four countries in the U.K. (England, Scotland, Northern Ireland, and Wales).[4]

Supplementary statistical analysis, handling of missing data, and power

For missing data, imputation replaced no more than 5% of missing values for a given metadatum with numeric values replaced with the median and categorical variables imputed using the mode. LHN, ADJ, DAD, and ATC had access to raw data. LHN performed data analysis, and the corresponding author had full access to data and the final responsibility to submit for publication. Based on the size of the assembled study population and the empirically observed vaccine hesitancy of 6%, we had 80% power to detect a minimum OR of 1.12 and 1.07, respectively, in the U.S. and the U.K. for risk of (or protection from) reporting vaccine hesitancy among non-white compared to white participants.

Supplementary Figures & Tables

Supplementary Figure 1. Study diagram

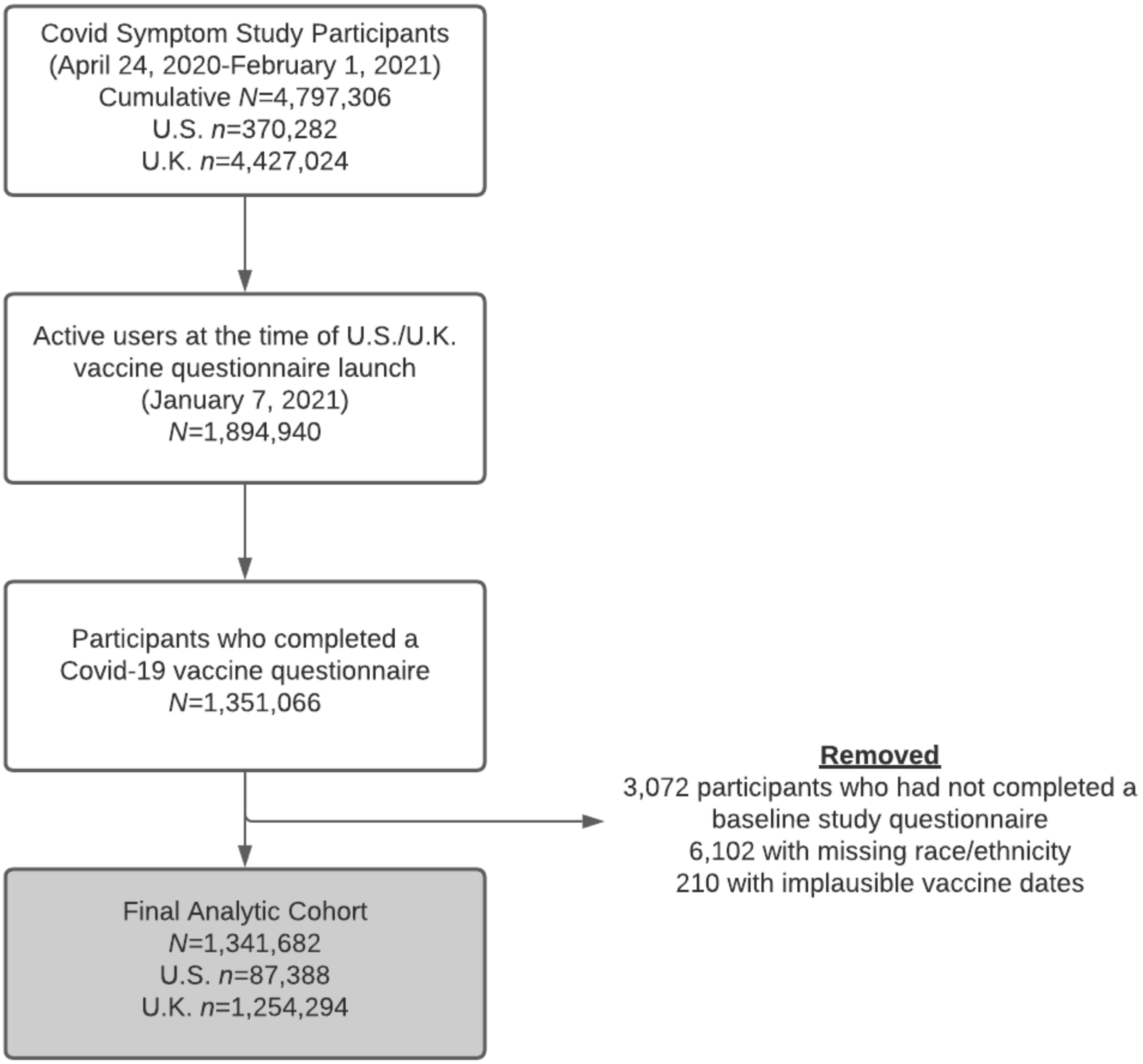

Supplementary Table 1. Baseline characteristics of study participants by race and ethnicity according to country of enrollment

	United States				
	White (*n*=76286)	Black (*n*=2468)	Hispanic (*n*=3838)	Asian (*n*=3602)	Other/More than one (*n*=1194)
**Age (years)**	60.9 (15.0)	58.5 (16.0)	50.5 (18.1)	55.8 (20.3)	56.9 (17.1)
<25	1804 (2.4)	110 (4.5)	341 (8.9)	370 (10.3)	58 (4.9)
25-34	4035 (5.3)	149 (6.0)	563 (14.7)	376 (10.4)	106 (8.9)
35-44	6603 (8.7)	210 (8.5)	597 (15.6)	409 (11.4)	136 (11.4)
45-54	8716 (11.4)	341 (13.8)	646 (16.8)	445 (12.4)	163 (13.7)
55-64	15970 (20.9)	635 (25.7)	664 (17.3)	434 (12.0)	237 (19.8)
≥65	39158 (51.3)	1023 (41.5)	1027 (26.8)	1568 (43.5)	494 (41.4)
**Female sex**	50523 (66.2)	1825 (73.9)	2421 (63.1)	2193 (60.9)	773 (64.7)
**BMI (kg/m^2)**	27.0 (6.0)	30.0 (7.0)	27.9 (6.4)	25.1 (5.1)	28. (6.7)
<18.5	1480 (1.9)	32 (1.3)	91 (2.4)	139 (3.9)	18 (1.5)
18.5-24.9	31208 (40.9)	582 (23.6)	1337 (34.8)	1900 (52.7)	404 (33.8)
25-29.9	25206 (33.0)	815 (33.0)	1261 (32.9)	1085 (30.1)	419 (35.1)
≥30	18392 (24.1)	1039 (42.1)	1149 (29.9)	478 (13.3)	353 (29.6)
**Comorbidities**					
Diabetes	3770 (4.9)	274 (11.1)	215 (5.6)	272 (7.6)	74 (6.2)
Heart Disease	5988 (7.8)	165 (6.7)	219 (5.7)	263 (7.3)	102 (8.5)
Lung Disease	2261 (3.0)	76 (3.1)	59 (1.5)	68 (1.9)	46 (3.9)

Kidney Disease	1392 (1.8)	63 (2.6)	65 (1.7)	79 (2.2)	37 (3.1)
Cancer	2202 (2.9)	55 (2.2)	80 (2.1)	79 (2.2)	27 (2.3)
**Education**	45.5 (18.4)	36.5 (17.5)	38.9 (18.9)	47.0 (17.7)	43.1 (19.1)
**Income**	81106 (31527)	68086 (28329)	74883 (30427)	89684 (31559)	78042 (30526)
**Current/prior smoking**	22254 (29.2)	723 (29.3)	992 (25.8)	838 (23.3)	392 (32.8)
**Healthcare worker**	5985 (7.8)	226 (9.2)	346 (9.0)	273 (7.6)	105 (8.8)
**Prior COVID-19**	4542 (6.0)	204 (8.3)	514 (13.4)	192 (5.3)	89 (7.5)

All racial categories were defined as each respective race not of Hispanic or Latino ancestry. Census-level data on education assessed the proportion of the general population above age 25 years with a bachelor's degree and income using median annual household income (U.S. dollars/year; **Supplementary Methods**). N (percentages) presented for categorical variables. Values are means (SD) for continuous variables. Values of polytomous variables may not sum to 100% due to rounding
Abbreviations: BMI (body mass index), m (meter), kg (kilogram).

	United Kingdom				
	White (*n*=1204721)	Black (*n*=9615)	South Asian (*n*=17628)	Middle East/East Asian (*n*=7689)	More than one/other (*n*=14641)
**Age (years)**	55.0 (15.4)	47.4 (15.6)	49.6 (15.1)	49.3 (15.4)	46.7 (15.7)
<25	46399 (3.9)	920 (9.6)	816 (4.6)	369 (4.8)	1462 (10.0)
25-34	92413 (7.7)	1312 (13.6)	2021 (11.5)	1055 (13.7)	2022 (13.8)
35-44	159125 (13.2)	1676 (17.4)	4063 (23.0)	1710 (22.2)	3122 (21.3)
45-54	238482 (19.8)	2186 (22.7)	4391 (24.9)	1668 (21.7)	3172 (21.7)
55-64	306131 (25.4)	2323 (24.2)	3145 (17.8)	1404 (18.3)	2811 (19.2)
≥65	362171 (30.1)	1198 (12.5)	3192 (18.1)	1483 (19.3)	2052 (14.0)
**Female sex**	711693 (59.1)	5642 (58.7)	9883 (56.1)	4438 (57.7)	9016 (61.6)
**BMI (kg/m^2)**	26.6 (6.0)	27.7 (7.2)	25.5 (5.7)	25.3 (6.0)	26.2 (6.7)
<18.5	31567 (2.6)	376 (3.9)	796 (4.5)	334 (4.3)	590 (4.0)
18.5-24.9	517674 (43.0)	3477 (36.2)	8779 (49.8)	4119 (53.6)	7033 (48.0)
25-29.9	404507 (33.6)	3087 (32.1)	5422 (30.8)	2131 (27.7)	4265 (29.1)
≥30	250973 (20.8)	2675 (27.8)	2631 (14.9)	1105 (14.4)	2753 (18.8)
**Comorbidities**					
Diabetes	39168 (3.3)	429 (4.5)	1233 (7.0)	275 (3.6)	461 (3.1)
Heart Disease	48828 (4.1)	258 (2.7)	832 (4.7)	300 (3.9)	440 (3.0)
Lung Disease	25971 (2.2)	155 (1.6)	294 (1.7)	118 (1.5)	226 (1.5)
Kidney Disease	11089 (0.9)	112 (1.2)	206 (1.2)	69 (0.9)	128 (0.9)
Cancer	17046 (2.1)	121 (1.8)	151 (1.3)	71 (1.4)	129 (1.4)

**Education**	7.1 (2.5)	6.5 (2.6)	7.24 (2.50)	7.5 (2.4)	7.4 (2.5)
**Income**	7.0 (2.5)	5.7 (2.7)	6.41 (2.65)	6.6 (2.6)	6.5 (2.6)
**Current/prior smoking**	272021 (22.6)	2210 (23.0)	2711 (15.4)	1533 (19.9)	3110 (21.2)
**Healthcare worker**	47300 (3.9)	636 (6.6)	1365 (7.7)	404 (5.3)	611 (4.2)
**Prior COVID-19**	72555 (6.0)	1020 (10.6)	1762 (10.0)	625 (8.1)	1242 (8.5)

Census-level data on education used the education score and income score for the Index of Multiple Deprivation, respectively **(Supplementary Methods)**. N (percentages) presented for categorical variables. Values are means (SD) for continuous variables. Values of polytomous variables may not sum to 100% due to rounding
Abbreviations: BMI (body mass index), m (meter), kg (kilogram)

Supplementary Table 2. Baseline characteristics of study participants by vaccine willingness and country of enrollment

	United States		
	**Willing** (*n*=67344)	**Unsure** (*n*=4943)	**Unwilling** (*n*=1363)
**Age (years)**	60.8 (15.5)	54.7 (17.2)	54.1 (16.8)
<25	2090 (3.1)	317 (6.4)	82 (6.0)
25-34	3681 (5.5)	486 (9.8)	145 (10.6)
35-44	5615 (8.3)	582 (11.8)	165 (12.1)
45-54	7548 (11.2)	783 (15.8)	217 (15.9)
55-64	13584 (20.2)	1063 (21.5)	320 (23.5)
≥65	34826 (51.7)	1712 (34.6)	434 (31.8)
**Female sex**	43181 (64.1)	3597 (72.8)	971 (71.2)
**Race/ethnicity**			
White	59429 (88.2)	3708 (75.0)	1007 (73.9)
Black	1568 (2.3)	468 (9.5)	143 (10.5)
Hispanic	2730 (4.1)	385 (7.8)	120 (8.8)
Asian	2780 (4.1)	262 (5.3)	47 (3.4)
More than one / other	837 (1.2)	120 (2.4)	46 (3.4)
**BMI (kg/m^2)**	27.0 (6.0)	28.0 (6.8)	27.9 (6.8)
<18.5	1372 (2.0)	121 (2.4)	36 (2.6)
18.5-24.9	27477 (40.8)	1792 (36.3)	502 (36.8)
25-29·9	22390 (33.2)	1493 (30.2)	405 (29.7)
≥30	16105 (23.9)	1537 (31.1)	420 (30.8)
**Comorbidities**			
Diabetes	3626 (5.4)	240 (4.9)	76 (5.6)
Heart Disease	5296 (7.9)	318 (6.4)	92 (6.7)
Lung Disease	1907 (2.8)	168 (3.4)	43 (3.2)
Kidney Disease	1290 (1.9)	97 (2.0)	34 (2.5)
Cancer	1955 (2.9)	113 (2.3)	19 (1.4)
**Education**	45.9 (18.4)	37.5 (17.5)	34.4 (16.3)

**Income**	81764 (31778)	73783 (28354)	70161 (25705)
**Current/prior smoking**	19582 (29.1)	1542 (31.2)	411 (30.2)
**Healthcare worker**	1833 (2.7)	301 (6.1)	153 (11.2)
**Prior COVID-19**	3734 (5.5)	702 (14.2)	263 (19.3)

All racial categories were defined as each respective race not of Hispanic or Latino ancestry. Census-level data on education assessed the proportion of the general population above age 25 years with a Bachelor's degree and income using median annual household income (U.S. dollars/year; **Supplementary Methods**). N (percentages) presented for categorical variables. Values are means (SD) for continuous variables. Values of polytomous variables may not sum to 100% due to rounding
Abbreviations: BMI (body mass index), m (meter), kg (kilogram)

	**United Kingdom**		
	**Willing** (*n*=1093110)	**Unsure** (*n*=52021)	**Unwilling** (*n*=9857)
**Age (years)**	54.9 (15.0)	46.1 (14.7)	45.0 (15.3)
<25	42646 (3.9)	4609 (8.9)	855 (8.7)
25-34	81363 (7.4)	7383 (14.2)	1907 (19.3)
35-44	142917 (13.1)	11521 (22.1)	2320 (23.5)
45-54	218009 (19.9)	12823 (24.6)	1979 (20.1)
55-64	285250 (26.1)	10027 (19.3)	1702 (17.3)
≥65	322925 (29.5)	5658 (10.9)	1094 (11.1)
**Female sex**	631477 (57.8)	35134 (67.5)	6467 (65.6)
**Race/ethnicity**			
White	1053810 (96.4)	47663 (91.6)	9071 (92.0)
Black	7171 (0.7)	1340 (2.6)	276 (2.8)
South Asian	13712 (1.3)	1258 (2.4)	229 (2.3)
Middle East/East Asian	6175 (0.6)	677 (1.3)	94 (1.0)
More than one/other	12242 (1.1)	1083 (2.1)	187 (1.9)
**BMI (kg/m^2)**	26.6 (6.0)	26.4 (6.4)	26.1 (6.4)
<18.5	28162 (2.6)	2122 (4.1)	478 (4.8)
18.5-24.9	470397 (43.0)	23423 (45.0)	4452 (45.2)
25-29.9	368595 (33.7)	15604 (30.0)	2972 (30.2)
≥30	225956 (20.7)	10872 (20.9)	1955 (19.8)
**Comorbidities**			

Diabetes	36909 (3.4)	1004 (1.9)	198 (2.0)
Heart Disease	42258 (3.9)	979 (1.9)	191 (1.9)
Lung Disease	22022 (2.0)	672 (1.3)	127 (1.3)
Kidney Disease	9459 (0.9)	315 (0.6)	61 (0.6)
Cancer	14540 (1.9)	311 (0.9)	88 (1.3)
**Index of multiple deprivation, education score**	7.1 (2.5)	6.5 (2.7)	6.4 (2.7)
**Index of multiple deprivation, income score**	7.0 (2.5)	6.4 (2.7)	6.3 (2.7)
**Current/prior smoking**	242643 (22.2)	12671 (24.4)	2404 (24.4)
**Healthcare worker**	24300 (2.2)	1972 (3.8)	653 (6.6)
**Prior COVID-19**	62681 (5.7)	4583 (8.8)	1034 (10.5)

All racial categories were defined as each respective race not of Hispanic or Latino ancestry. Census-level data on education used the education score and income score for the Index of Multiple Deprivation, respectively (**Supplementary Methods**). N (percentages) presented for categorical variables. Values are means (SD) for continuous variables. Values of polytomous variables may not sum to 100% due to rounding
Abbreviations: BMI (body mass index), m (meter), kg (kilogram)

Supplementary Table 3. Inverse probability weighted analyses of vaccine hesitancy and uptake by race and ethnicity according to country of enrollment

	United States				
	White	Black	Hispanic	Asian	More than one/other
**Vaccine hesitancy**					
**Number hesitant or unsure / total**	4715/64144	611/2179	505/3235	309/3089	166/1003
**Multivariable-adjusted OR (95% CI)[1]**	1.0 (ref.)	2.90 (2.58 to 3.38)	1.48 (1.30 to 1.69)	1.06 (0.89 to 1.26)	1.98 (1.54 to 2.54)
**Vaccine uptake (all)**					
**Number receiving a vaccine / total**	15341/64144	362/2179	519/3235	716/3089	202/1003
**Multivariable-adjusted OR (95% CI)[1]**	1.0 (ref.)	0.70 (0.60 to 0.81)	0.96 (0.86 to 1.07)	1.03 (0.92 to 1.14)	1.04 (0.84 to 1.28)
**Vaccine uptake (vaccine willing)**					
**Number receiving a vaccine / total**	15062/59429	328/1568	499/2730	681/2780	192/837
**Multivariable-adjusted OR (95% CI)[1]**	1.0 (ref.)	0.83 (0.71 to 0.97)	0.98 (0.88 to 1.09)	1.00 (0.90 to 1.12)	1.10 (0.88 to 1.38)

	United Kingdom				
	White	Black	South Asian	Middle East/East Asian	More than one/other
**Vaccine hesitancy**					
**Number hesitant or unsure / total**	567734/1110544	1616/8787	1487/15199	771/6946	1270/13512
**Multivariable-adjusted OR (95% CI)[1]**	1.0 (ref.)	2.52 (2.35 to 2.70)	1.52 (1.39 to 1.66)	1.58 (1.40 to 1.79)	1.40 (1.30 to 1.52)
**Vaccine uptake (all)**					
**Number receiving a vaccine / total**	171453/1110544	1022/8787	2339/15199	922/6946	1506/13512
**Multivariable-adjusted OR (95% CI)[1]**	1.0 (ref.)	0.93 (0.86 to 1.01)	1.22 (1.16 to 1.28)	1.06 (0.98 to 1.16)	1.03 (0.97 to 1.09)
**Vaccine uptake (vaccine willing)**					

**Number receiving a vaccine / total**	168369/1053810	951/7171	2255/13712	884/6175	1469/12242
**Multivariable-adjusted OR (95% CI)[1]**	1.0 (ref.)	1.01 (0.93 to 1.10)	1.24 (1.18 to 1.31)	1.08 (0.99 to 1.18)	1.06 (1.00 to 1.13)

[1]Conditioned upon age and date of study entry, adjustment for personal history of diabetes, heart disease, lung disease, kidney disease, current smoking status, body mass index, prior reported history of COVID-19 infection, frontline healthcare worker status, region, and education and income at the community level with inverse probability weighting
Abbreviations: CI (confidence interval), OR (odds ratio)

Supplementary Table 4. Vaccine hesitancy by geographical region according to country of enrollment

	United States			
	Northeast	Midwest	South	West
**Number of individuals hesitant or unsure / total**	1174/16365	1133/13093	1931/17633	1969/24474
**Age-adjusted OR (95% CI)**[1]	1.0 (ref.)	1.16 (1.06 to 1.26)	1.43 (1.33 to 1.54)	1.16 (1.08 to 1.25)
**Multivariable-adjusted OR (95% CI)**[2]	1.0 (ref.)	1.12 (1.03 to 1.23)	1.31 (1.21 to 1.41)	1.13 (1.05 to 1.23)
**Multivariable-adjusted OR (95% CI)**[3]	1.0 (ref.)	1.03 (0.94 to 1.12)	1.24 (1.14 to 1.34)	1.04 (0.96 to 1.12)

[1]Conditioned upon age and date of study entry
[2]Additional conditioning upon sex and adjustment for race/ethnicity, personal history of diabetes, heart disease, lung disease, kidney disease, current smoking status, body mass index, and prior reported history of COVID-19 infection
[3]Additional adjustment for frontline healthcare worker status and education and income at the community level

Regions defined by the U.S. Census Bureau guidelines. The Northeast included the states of Connecticut, Maine, Massachusetts, New Hampshire, New Jersey, New York, Pennsylvania, Rhode Island, and Vermont. The Midwest included the states of Illinois, Indiana, Iowa, Kansas, Michigan, Minnesota, Missouri, Nebraska, North Dakota, Ohio, South Dakota, and Wisconsin. The South included Alabama, Arkansas, Delaware, Florida, Georgia, Kentucky, Louisiana, Maryland, Mississippi, North Carolina, Oklahoma, South Carolina, Tennessee, Texas, Virginia, and West Virginia. The West included Alaska, Arizona, California, Colorado, Hawaii, Idaho, Montana, New Mexico, Nevada, Oregon, Utah, Washington, and Wyoming.

Abbreviations: CI (confidence interval), OR (odds ratio)

	United Kingdom			
	England	Northern Ireland	Scotland	Wales
**Number of individuals hesitant or unsure / total**	50580/952267	434/5994	2744/56232	2581/49811
**Age-adjusted OR (95% CI)**[1]	1.0 (ref.)	1.38 (1.25 to 1.51)	0.96 (0.93 to 1.00)	1.10 (1.06 to 1.15)
**Multivariable-adjusted OR (95% CI)**[2]	1.0 (ref.)	1.44 (1.31 to 1.58)	1.00 (0.96 to 1.04)	1.13 (1.08 to 1.18)
**Multivariable-adjusted OR (95% CI)**[3]	1.0 (ref.)	1.42 (1.30 to 1.57)	1.00 (0.96 to 1.04)	1.11 (1.07 to 1.16)

[1]Conditioned upon age and date of study entry
[2]Additional conditioning upon sex and adjustment for race/ethnicity, personal history of diabetes, heart disease, lung disease, kidney disease, current smoking status, body mass index, and prior reported history of COVID-19 infection
[3]Additional adjustment for frontline healthcare worker status and education and income at the community level

Abbreviations: CI (confidence interval), OR (odds ratio)

Supplementary Table 5. Reasons for vaccine hesitancy by race and ethnicity according to country of enrollment

Reason Cited (%)	United States				
	White (*n*=4715)	Black (*n*=611)	Hispanic (*n*=505)	Asian (*n*=309)	More than one/other (*n*=166)
Concerned about the long-term side effects	51.8	57.3	54.9	49.5	47.0
Concerned about adverse reaction	44.7	54.2	51.9	47.2	45.8
Do not know enough about it	41.7	50.6	50.9	42.4	41.6
Personal belief/philosophical reasons	8.8	14.1	13.1	7.8	10.8
Illness/Medication	8.3	8.7	7.5	7.1	12.1
Do not think it is necessary	6.7	2.5	4.2	3.9	10.8
Do not think it will be available to me	5.0	4.6	6.5	7.1	6.0
Prefer not to say	4.3	6.2	4.2	4.2	4.2
Do not think it will work	3.9	3.8	4.6	2.9	7.2
Pregnancy/breastfeeding	2.4	0.7	2.8	4.5	1.2
Religious reasons	1.9	1.3	3.6	2.6	2.4
I took part in a vaccine trial	0.3	0.3	0.4	0.3	0.6
Other	9.8	9.8	6.1	9.4	13.9

Proportions are calculated within racial and ethnic categories among individuals expressing vaccine unwillingness or unsure if they will obtain one. Participants were allowed to check more than one reason.

Reason Cited (%)	United Kingdom				
	White (*n*=56775)	Black (*n*=1617)	South Asian (*n*=1487)	Middle East/East Asian (*n*=771)	More than one/other (*n*=1270)
Concerned about the long-term side effects	49.9	52.9	50.9	55.6	51.3
Concerned about adverse reaction	30.2	39.3	36.5	37.2	34.4
Do not know enough about it	36.7	45.0	41.9	39.8	36.0

Personal belief/philosophical reasons	5.4	7.3	6.3	4.0	5.0
Illness/Medication	5.8	5.9	5.5	5.3	4.9
Do not think it is necessary	7.5	5.6	5.9	5.1	7.6
Do not think it will be available to me	9.0	7.2	9.8	8.4	10.9
Prefer not to say	5.8	8.0	8.3	6.2	5.5
Do not think it will work	3.0	2.8	4.7	3.4	3.7
Pregnancy/breastfeeding	7.6	4.8	5.9	6.0	6.3
Religious reasons	0.5	1.9	1.8	1.0	1.0
I took part in a vaccine trial	0.4	0.1	0.4	0.4	0.3
Other	8.2	6.2	5.1	7.4	9.0

Proportions are calculated within racial and ethnic categories among individuals expressing vaccine unwillingness or unsure if they will obtain one. Participants were allowed to check more than one reason.

Supplementary Table 6. Baseline characteristics of study participants by vaccination status and country of enrollment

	United States	
	**Vaccinated** (*n*=17140)	**Unvaccinated** (*n*=56,510)
**Age (years)**	69.0 (11.9)	57.6 (15.8)
<25	86 (0.5)	2403 (4.3)
25-34	369 (2.2)	3943 (7.0)
35-44	577 (3.4)	5785 (10.2)
45-54	809 (4.7)	7739 (13.7)
55-64	1674 (9.8)	13293 (23.5)
≥65	13625 (79.5)	23347 (41.3)
**Female sex**	11325 (66.1)	36424 (64.5)
**Race/ethnicity**		
White	15341 (89.5)	48803 (86.4)
Black	362 (2.1)	1817 (3.2)
Hispanic	519 (3.0)	2716 (4.8)
Asian	716 (4.2)	2373 (4.2)
More than one / other	202 (1.2)	801 (1.4)
**BMI (kg/m^2)**	26.6 (5.6)	27.2 (6.2)
<18.5	7258 (42.3)	22513 (39.8)
18.5-24.9	350 (2.0)	1179 (2.1)
25-29.9	5980 (34.9)	18308 (32.4)
≥30	3552 (20.7)	14510 (25.7)
**Comorbidities**		
Diabetes	1157 (6.8)	2785 (4.9)
Heart Disease	2030 (11.8)	3676 (6.5)
Lung Disease	640 (3.7)	1478 (2.6)
Kidney Disease	444 (2.6)	977 (1.7)
Cancer	718 (4.2)	1369 (2.4)
**Education**	45.2 (18.2)	45.0 (18.7)

**Income**	81071 (31280)	80969 (31710)
**Current/prior smoking**	5745 (33.5)	15790 (27.9)
**Healthcare worker**	955 (5.6)	1332 (2.4)
**Prior COVID-19**	499 (2.9)	4200 (7.4)

All racial categories categories were defined as each respective race not of Hispanic or Latino ancestry. Census-level data on education assessed the proportion of the general population above age 25 years with a Bachelor's degree and income using median annual household income. N (percentages) presented for categorical variables. Values are means (SD) for continuous variables. Values of polytomous variables may not sum to 100% due to rounding
Abbreviations: BMI (body mass index), m (meter), kg (kilogram)

	**United Kingdom**	
	**Vaccinated** (*n*=177242)	**Unvaccinated** (*n*=977746)
**Age (years)**	65.9 (14.6)	52.3 (14.3)
<25	1634 (0.9)	46476 (4.8)
25-34	6095 (3.4)	84558 (8.6)
35-44	11012 (6.2)	145746 (14.9)
45-54	19009 (10.7)	213802 (21.9)
55-64	27574 (15.6)	269405 (27.6)
≥65	111918 (63.1)	217759 (22.3)
**Female sex**	108311 (61.1)	564767 (57.8)
**Race/ethnicity**		
White	171453 (96.7)	939091 (96.0)
Black	1022 (0.6)	7765 (0.8)
South Asian	2339 (1.3)	12860 (1.3)
Middle East/East Asian	922 (0.5)	6024 (0.6)
More than one/other	1506 (0.8)	12006 (1.2)
**BMI (kg/m^2)**	26.6 (5.9)	26.6 (6.1)
<18.5	77265 (43.6)	421007 (43.1)
18.5-24.9	4840 (2.7)	25922 (2.7)
25-29·9	61296 (34.6)	325875 (33.3)
≥30	33841 (19.1)	204942 (21.0)
**Comorbidities**		

Diabetes	9125 (5.1)	28986 (3.0)
Heart Disease	15038 (8.5)	28390 (2.9)
Lung Disease	8461 (4.8)	14360 (1.5)
Kidney Disease	3221 (1.8)	6614 (0.7)
Cancer	5969 (4.6)	8970 (1.4)
**Education**	7.2 (2.5)	7.0 (2.5)
**Income**	7.1 (2.4)	6.9 (2.5)
**Current/prior smoking**	45240 (25.5)	212478 (21.7)
**Healthcare worker**	15684 (8.8)	11241 (1.1)
**Prior COVID-19**	8771 (4.9)	59527 (6.1)

All racial categories were defined as each respective race not of Hispanic or Latino ancestry. Census-level data on education used the education score and income score for the Index of Multiple Deprivation ,respectively. N (percentages) presented for categorical variables. Values are means (SD) for continuous variables. Values of polytomous variables may not sum to 100% due to rounding
Abbreviations: BMI (body mass index), m (meter), kg (kilogram)

Supplementary Table 7. Vaccine uptake by geographical region according to country of enrollment

	United States			
	Northeast	Midwest	South	West
**Number of individuals receiving a vaccine / total**	2942/16365	2569/13093	4541/17633	6748/24474
**Age-adjusted OR (95% CI)[1]**	1.0 (ref.)	1.13 (1.07 to 1.19)	1.69 (1.61 to 1.77)	1.52 (1.46 to 1.59)
**Multivariable-adjusted OR (95% CI)[2]**	1.0 (ref.)	1.14 (1.08 to 1.21)	1.70 (1.62 to 1.79)	1.52 (1.45 to 1.59)
**Multivariable-adjusted OR (95% CI)[3]**	1.0 (ref.)	1.14 (1.08 to 1.21)	1.73 (1.65 to 1.82)	1.55 (1.48 to 1.62)

[1]Conditioned upon age and date of study entry
[2]Additional conditioning upon sex and adjustment for race/ethnicity, personal history of diabetes, heart disease, lung disease, kidney disease, current smoking status, body mass index, and prior reported history of COVID-19 infection
[3]Additional adjustment for frontline healthcare worker status and education and income at the community level

Regions defined by the U.S. Census Bureau guidelines. The Northeast included the states of Connecticut, Maine, Massachusetts, New Hampshire, New Jersey, New York, Pennsylvania, Rhode Island, and Vermont. The Midwest included the states of Illinois, Indiana, Iowa, Kansas, Michigan, Minnesota, Missouri, Nebraska, North Dakota, Ohio, South Dakota, and Wisconsin. The South included Alabama, Arkansas, Delaware, Florida, Georgia, Kentucky, Louisiana, Maryland, Mississippi, North Carolina, Oklahoma, South Carolina, Tennessee, Texas, Virginia, and West Virginia. The West included Alaska, Arizona, California, Colorado, Hawaii, Idaho, Montana, New Mexico, Nevada, Oregon, Utah, Washington, and Wyoming.

Abbreviations: CI (confidence interval), OR (odds ratio). Data shown through February 1, 2021.

	United Kingdom			
	England	Northern Ireland	Scotland	Wales
**Number of individuals receiving a vaccine / total**	151200/952267	884/5994	4290/56232	6770/49811
**Age-adjusted OR (95% CI)[1]**	1.0 (ref.)	0.92 (0.86 to 0.98)	0.42 (0.40 to 0.43)	0.72 (0.70 to 0.74)
**Multivariable-adjusted OR (95% CI)[2]**	1.0 (ref.)	0.91 (0.85 to 0.97)	0.42 (0.40 to 0.42)	0.72 (0.70 to 0.73)
**Multivariable-adjusted OR (95% CI)[3]**	1.0 (ref.)	0.89 (0.83 to 0.95)	0.41 (0.40 to 0.42)	0.71 (0.70 to 0.73)

[1]Conditioned upon age and date of study entry
[2]Additional conditioning upon sex and adjustment for race/ethnicity, personal history of diabetes, heart disease, lung disease, kidney disease, current smoking status, body mass index, and prior reported history of COVID-19 infection
[3]Additional adjustment for frontline healthcare worker status and education and income at the community level

Abbreviations: CI (confidence interval), OR (odds ratio). Data shown through February 1, 2021.

Supplementary Table 8. Localized symptoms among vaccinated participants according to country of enrollment

	United States				
	White (*n*=32316)	Black (*n*=811)	Hispanic (*n*=1198)	Asian (*n*=1328)	More than one/other (*n*=431)
Pain	9115 (28.2)	228 (28.1)	400 (33.4)	492 (37.0)	134 (31.1)
Redness	708 (2.2)	19 (2.3)	36 (3.0)	30 (2.3)	12 (2.8)
Swelling	1427 (4.4)	73 (9.0)	78 (6.5)	98 (7.4)	27 (6.3)
Swollen lymph nodes	159 (0.5)	11 (1.4)	13 (1.1)	6 (0.5)	4 (0.9)
Warmth	1463 (4.5)	46 (5.7)	94 (7.8)	76 (5.7)	38 (8.8)
Bruising	78 (1.1)	1 (0.5)	10 (4.6)	3 (1.4)	2 (2.1)
Itchiness	457 (1.4)	27 (3.3)	22 (1.8)	29 (2.2)	4 (0.9)
Tenderness	11950 (37.0)	281 (34.6)	466 (38.9)	528 (39.8)	157 (36.4)

	United Kingdom				
	White (*n*=393456)	Black (*n*=2218)	South Asian (*n*=5092)	Middle East/East Asian (*n*=2051)	More than one/other (*n*=3378)
Pain	53666 (13.6)	594 (26.8)	1662 (32.6)	514 (25.1)	800 (23.7)
Redness	6387 (1.6)	56 (2.5)	131 (2.6)	45 (2.2)	82 (2.4)
Swelling	10144 (2.6)	158 (7.1)	302 (5.9)	81 (3.9)	160 (4.7)
Swollen lymph nodes	1558 (0.4)	23 (1.0)	46 (0.9)	11 (0.5)	22 (0.7)
Warmth	15787 (4.0)	164 (7.4)	300 (5.9)	119 (5.8)	171 (5.1)
Bruising	2095 (1.7)	12 (2.0)	18 (1.5)	12 (2.2)	25 (2.6)
Itchiness	4029 (1.0)	38 (1.7)	78 (1.5)	29 (1.4)	58 (1.7)
Tenderness	111833 (28.4)	760 (34.3)	1559 (30.6)	592 (28.9)	1101 (32.6)

Proportions are calculated within racial and ethnic categories among individuals expressing vaccine unwillingness or unsure if they will obtain one. Participants were allowed to check more than one reason.

Supplementary Table 9. Vaccine-related questions through February 1, 2021

Vaccine Willingness

Would you accept a COVID-19 vaccine if offered?

1. Yes
2. No
3. Unsure

If No or Unsure, tell us why. (Tick all that apply)

1. I took part in a vaccine trial
2. Religious reasons
3. Personal belief/philosophical reasons
4. Pregnancy/breastfeeding
5. Concerned about the long-term side effects
6. Do not know enough about it
7. Illness/Medication
8. Do not think it will be available to me
9. Do not think it is necessary
10. Do not think it will work
11. Concerned about adverse reaction
12. Prefer not to say
13. Other (free text)

Vaccine Uptake

Vaccine brand

Dose number

Date received

Batch (U.S.)/Lot (U.K.) number

Vaccine-related Symptoms

Are you experiencing any symptoms near the injection site? (Check all that apply)

1. Pain
2. Redness
3. Swelling
4. Swollen glands in the armpit
5. Warmth
6. Itch
7. Tenderness
8. Bruising
9. Other (free text)

Supplementary Table 10. Race and ethnicity categories by country of enrollment

United States	
**Responses**	**Categories**
Non-Hispanic white	White, non-Hispanic
Black or African-American	Black
Hispanic/LatinX	Hispanic
Asian	Asian
Native Hawaiian or other Pacific Islander	Asian
American Indian or Alaska Native	More than one/other
Other non-Hispanic racial group, other racial or ethnic group, multiple race/ethnicity groups identified	More than one/other

United Kingdom	
**Responses**	**Categories**
White- British, Irish, or other	White
Black/Black British - Caribbean, African, or other	Black
Asian/Asian British - Indian, Pakistani, Bangladeshi, or other	South Asian
Chinese/Chinese British	Middle East/East Asian
Middle Eastern/Middle Eastern British - Arab, Turkish, other	Middle East/East Asian
East/Southeast Asian, other, or mixed/multiple race - white and Black/Black British, other	More than one/other

Supplementary Table 11. Partnering cohorts within the COPE Consortium

Cohort Name	Cohort contact (non-contributing authors)
ACS Cancer Prevention Study 3	Alpa V. Patel, Anusila Deka
The Agricultural Health Study	Laura Beane-Freeman/ Dale P Sandler
ASPREE-XT	Michael E. Ernst, Brenda Kirpach, Anne M. Murray
Black Women's Health Study	Julie R. Palmer, Lynn Rosenberg
Growing Up to Today Study	Jaime E. Hart, Jorge E. Chavarro
The GuLF Study	Dale P. Sandler
Health Professionals Follow-up Study	Lorelei A. Mucci, Walter C. Willett
Multiethnic Cohort Study	Loic Le Marchand, Lynne R. Wilkens
Nurses' Health Study I, II and 3	Meir Stampfer, Walter C. Willett, Heather A. Eliassen, Jaime E. Hart, Jorge E. Chavarro
PREDICT 2	NA
The Sister Study	Dale P. Sandler
Stanford Nutrition Studies	Christopher D. Gardner
UCSD/COH California Teachers Study	Maria Elena Martinez, James V. Lacey
Twins U.K.	NA

Supplementary References

1. Download Data [Internet]. [cited 2021 Feb 14];Available from: https://www.ers.usda.gov/data-products/county-level-data-sets/download-data/

2. Website [Internet]. [cited 2021 Feb 14];Available from: https://www.gov.uk/government/statistics/english-indices-of-deprivation-2019

3. Census geography [Internet]. [cited 2021 Feb 18];Available from: https://www.ons.gov.uk/methodology/geography/ukgeographies/censusgeography

4. Census geography [Internet]. [cited 2021 Feb 18];Available from: https://www.ons.gov.uk/methodology/geography/ukgeographies/censusgeography
