# Supplementary material for: Self-reported COVID-19 vaccine hesitancy and uptake among participants from different racial and ethnic groups in the United States and United Kingdom
Source: Nat Commun. 2022 Feb 1;13:636. doi: 10.1038/s41467-022-28200-3 (PMC8807721; doi:10.1038/s41467-022-28200-3)
Supplement: Supplementary file 2 — Reporting Summary [file 41467_2022_28200_MOESM2_ESM.pdf]

nature portfolio

Corresponding author(s): Andrew T. Chan

Last updated by author(s): 1/11/2022

Reporting Summary

Nature Portfolio wishes to improve the reproducibility of the work that we publish. This form provides structure for consistency and transparency in reporting. For further information on Nature Portfolio policies, see our Editorial Policies and the Editorial Policy Checklist.

Statistics

For all statistical analyses, confirm that the following items are present in the figure legend, table legend, main text, or Methods section.

n/a	Confirmed	
☐	☒	The exact sample size (*n*) for each experimental group/condition, given as a discrete number and unit of measurement
☐	☒	A statement on whether measurements were taken from distinct samples or whether the same sample was measured repeatedly
☐	☒	The statistical test(s) used AND whether they are one- or two-sided<br>*Only common tests should be described solely by name; describe more complex techniques in the Methods section.*
☐	☒	A description of all covariates tested
☒	☐	A description of any assumptions or corrections, such as tests of normality and adjustment for multiple comparisons
☐	☒	A full description of the statistical parameters including central tendency (e.g. means) or other basic estimates (e.g. regression coefficient) AND variation (e.g. standard deviation) or associated estimates of uncertainty (e.g. confidence intervals)
☐	☒	For null hypothesis testing, the test statistic (e.g. *F*, *t*, *r*) with confidence intervals, effect sizes, degrees of freedom and *P* value noted<br>*Give P values as exact values whenever suitable.*
☒	☐	For Bayesian analysis, information on the choice of priors and Markov chain Monte Carlo settings
☒	☐	For hierarchical and complex designs, identification of the appropriate level for tests and full reporting of outcomes
☐	☒	Estimates of effect sizes (e.g. Cohen's *d*, Pearson's *r*), indicating how they were calculated

Our web collection on statistics for biologists contains articles on many of the points above.

Software and code

Policy information about availability of computer code

Data collection	Data was collected using the COVID Symptom Study smartphone application, as retrieved in February 2021. The app is freely available mobile software developed by Zoe Global Ltd. in collaboration with researchers and clinicians at King's college London and Massachusetts General Hospital. Code for data extraction is available at https://github/com/KCL-BMEIS/ExeTera.
Data analysis	All statistical analyses were performed using R 4.0.3 (Vienna, Austria) and packages from the Bioconductor 3.12 release.

For manuscripts utilizing custom algorithms or software that are central to the research but not yet described in published literature, software must be made available to editors and reviewers. We strongly encourage code deposition in a community repository (e.g. GitHub). See the Nature Portfolio guidelines for submitting code & software for further information.

Data

Policy information about availability of data

All manuscripts must include a data availability statement. This statement should provide the following information, where applicable:

- Accession codes, unique identifiers, or web links for publicly available datasets
- A description of any restrictions on data availability
- For clinical datasets or third party data, please ensure that the statement adheres to our policy

All study authors had access to participant-level data. To comply with informed participant consent, which stipulated raw anonymized individual-level data would only be available upon request for research purposes, data collected using the COVID Symptom Study smartphone application are being shared with other researchers through the U.K. National Health Service-funded Health Data Research UK (HDRUK) and Secure Anonymised Information Linkage consortium, housed in the U.K. Secure Research Platform (Swansea, UK, https://web.www.healthdatagateway.org/dataset/fddcb382-3051-4394-8436-b92295f14259). U.S. investigators are encouraged to coordinate data requests through the Coronavirus Pandemic Epidemiology (COPE) Consortium (Suppl. Table 11, https://

www.monganinstitute.org/cope-consortium). Aggregated source data are provided with this paper.

Field-specific reporting

Please select the one below that is the best fit for your research. If you are not sure, read the appropriate sections before making your selection.

- [x] Life sciences
- [ ] Behavioural & social sciences
- [ ] Ecological, evolutionary & environmental sciences

For a reference copy of the document with all sections, see nature.com/documents/nr-reporting-summary-flat.pdf

Life sciences study design

All studies must disclose on these points even when the disclosure is negative.

Sample size	From March 24, 2020 to February 1, 2021, we enrolled a total of 4,797,306 individuals (n=370,282 U.S. participants and n=4,427,024 U.K participants), of whom 1,894,940 individuals were active/logged at least one entry in Dec 2020 (i.e., two weeks prior to the initial vaccine questionnaire). After excluding participants who did not provide their racial/ethnic information and restricting to those who responded to at least one vaccine questionnaires, a final analytic cohort of 1,341,682 individuals remained). Sample size was not determined a priori, and we enrolled all consecutive eligible patients within the study period.
Data exclusions	We excluded participants who did not provide their racial/ethnic information or did not respond to at least one vaccine questionnaire (pre-established).
Replication	This is an observational study using data from two different countries (the U.S. and U.K.) which were analyzed separately.
Randomization	Allocation of the primary exposure (race/ethnicity) is not possible, though multivariable adjustment included age, date of study entry, sex, personal history of diabetes, heart disease, lung disease, kidney disease, current smoking status, body mass index, prior reported history of COVID-19 infection, frontline healthcare worker status, region, and education and income at the community level.
Blinding	Blinding based on primary exposure (race/ethnicity) is not applicable to this study as race/ethnicity were self-reported (and thus, not blinded).

Reporting for specific materials, systems and methods

We require information from authors about some types of materials, experimental systems and methods used in many studies. Here, indicate whether each material, system or method listed is relevant to your study. If you are not sure if a list item applies to your research, read the appropriate section before selecting a response.

Materials & experimental systems

n/a	Involved in the study	
x		Antibodies
x		Eukaryotic cell lines
x		Palaeontology and archaeology
x		Animals and other organisms
	x	Human research participants
	x	Clinical data
x		Dual use research of concern

Methods

n/a	Involved in the study	
x		ChIP-seq
x		Flow cytometry
x		MRI-based neuroimaging

Human research participants

Policy information about studies involving human research participants

Population characteristics	Baseline population characteristics have been summarized in Table 1 and Supplementary Table 1.
Recruitment	Our study population includes all participants enrolled in the COVID Symptom Study smartphone application as described above (March 24, 2020 to February 1, 2021) in the United States and United Kingdom. Participants were recruited through general and social media outreach, as well as direct invitations from the investigators of long-running prospective cohorts to study participants. All participants had access to smartphone technology which could have impacted our results.
Ethics oversight	Mass General Brigham Human Research Committee (Institutional Review Board Protocol 2020P000909) and King's College London Ethics Committee (REMAS ID 18210)

Note that full information on the approval of the study protocol must also be provided in the manuscript.

Clinical data

Policy information about clinical studies

All manuscripts should comply with the ICMJEguidelines for publication of clinical research and a completedCONSORT checklist must be included with all submissions.

Clinical trial registration	The original protocol detailing an observational study to develop a symptom-based classifier is registered with ClinicalTrials.gov (NCT04331509). The CSS application was later adapted to capture data additional unregistered outcomes. The co-primary outcomes of an additional vaccine questionnaire were vaccine hesitancy and uptake.
Study protocol	The full protocol may be requested from the respective Ethics oversight committees [Mass General Brigham Human Research Committee (Institutional Review Board Protocol 2020P000909) and King's College London Ethics Committee (REMAS ID 18210)].
Data collection	Our study population includes all participants enrolled in the COVID Symptom Study smartphone application as described above . Participants were recruited through general and social media outreach, as well as direct invitations from the investigators of long-running prospective cohorts to study participants.
Outcomes	This study's co-primary outcomes are vaccine hesitancy and vaccine uptake, which were determined a priori, and assessed using vaccine hesitancy and uptake questionnaires.
